# A CLINICAL TRIAL USING METHYLATION AGE TO EVALUATE CURRENT ANTIAGING PRACTICES

**DOI:** 10.1093/geroni/igac059.2723

**Published:** 2022-12-20

**Authors:** Josh Mitteldorf

**Affiliations:** DataBETA Project, Philadelphia, Pennsylvania, United States

## Abstract

Recent advances in the technology of ‘‘aging clocks’’ based on DNA methylation suggest that it may be possible to measure changes in the rate of human aging over periods as short as a year or two. To the extent that methylation (and other biomarkers) are valid surrogates for biological age, the testing of antiaging interventions has thus become radically cheaper, faster, and more practical. Together with colleagues at McGill University, I have initiated a clinical trial to evaluate some of the most popular antiaging strategies currently deployed by ‘‘early adopters’’ in the lay community of personal health activists. We are recruiting 5000 subjects, age 45–65, and interviewing them in detail about their diet, drugs and supplements, exercise, social, and other practices that plausibly contribute to modulate the rate of aging. They agree to submit saliva samples for analysis of methylation age at the beginning and end of a 2-year test period. Primary endpoint is the difference in methylation age over the course of 2 years. Results will be viewed as an exploratory study to identify synergistic combinations of age-retarding treatments. All data (redacted for privacy) will be open sourced, available to the scientific community and to the public.

